# Neuromyelitis optica and concomitant pulmonary tuberculosis: a case report 

**DOI:** 10.1186/s13256-021-03002-1

**Published:** 2021-08-06

**Authors:** Zakaria Saied, Fatma Nabli, Amine Rachdi, Cyrine Jeridi, Bissene Douma, Samir Belal, Samia Ben Sassi

**Affiliations:** Neurology Department, Mongi Ben Hmida National Institute of Neurology, University of Tunis El Manar, Jbel Lakhdhar Street 1007, LaRabta Bab Saâdoun, Tunis, Tunisia

**Keywords:** Neuromyelitis optica, Pulmonary tuberculosis, Optic neuritis, Acute transverse myelitis

## Abstract

**Background:**

Concomitant diagnosis of neuromyelitis optica spectrum disease and pulmonary tuberculosis has rarely been reported.

**Case report:**

We report a case involving a young Tunisian male patient who developed dry cough followed, 2 months later, by weakness in the lower limbs. The findings of central nervous system imaging and anti-aquaporin-4 antibody positivity were compatible with the diagnosis of neuromyelitis optica spectrum disease. Constellation of the clinical and the typical radiological pulmonary findings in our patient, coming from an endemic region, allowed the diagnosis of pulmonary tuberculosis, although sputum smear examination for acid-fast bacilli and cultures was negative. The patient received anti-tuberculous polytherapy associated with immunomodulation, consisting of methylprednisolone and intravenous immunoglobulins. Pulmonary infection symptoms initially improved but with no motor recovery. The patient suddenly died at home 4 months after the onset of the first symptoms. Current data regarding the clinical presentation of this underreported concomitant or associated condition, the possible pathophysiological mechanisms, and the therapeutic options were reviewed.

**Conclusions:**

This case underscores the necessity to understand the exact mechanism of these coincident entities and to clarify the best immunomodulatory choice since immunosuppression targeting neuromyelitis optica spectrum disease can lead to dissemination of pulmonary tuberculosis.

## Introduction

Neuromyelitis optica spectrum disease (NMOSD) is an autoimmune inflammatory disease affecting the central nervous system. Variations in prevalence have been described among different geographic areas and ethnicities [1]. Prevalence estimates range from 0.34 to 10/100,000 in adults [1].The frequency of NMOSD diagnosis was 5.3 % in 170 Tunisian patients with a context of optic neuritis (ON) and/or myelitis [2]. Six core clinical characteristics are identified in NMOSD, including optic neuritis, acute myelitis (mainly longitudinally extensive transverse myelitis, LETM), area postrema syndrome, acute brainstem, and diencephalic or cerebral syndromes. Most patients with NMOSD have positive serum immunoglobulin G (IgG) antibodies targeting the water channel aquaporin-4 [3]. Anti-aquaporin-4 autoantibodies (anti-AQP4ab) are specific markers of the disease. They react with the aquaporin in the astrocytes end-feet, thus inducing complement activation [4, 5]. NMOSD affects young adults with a female predominance. Extra pulmonary tuberculosis dissemination, including the nervous system, passes through hematogenous dissemination. After bacteremia, *Mycobacterium tuberculosis* (MT) crosses the blood–brain barrier and scattered foci develop into the central nervous system [6, 7]. The foci trigger an inflammatory reaction in a susceptible host [8] and remain quiescent. Subsequently, at the moment of immunodepression, the rupture of foci induces tuberculoma, bacterial encephalitis, meningitis, or vasculopathy [6, 7].

After the discovery of anti-AQP4ab, a small number of reports, involving diagnoses of concomitant [5, 9, 10] or associated [4, 11, 12] NMOSD and pulmonary tuberculosis (PT) have been published. For associated NMOSD and PT, the neurological symptoms attributed to both the central nervous system (CNS) due to NMOSD and to PT dissemination into CNS defined the conditions. PT symptoms often shortly precede NMOSD manifestations [5, 9, 12], suggesting that *Mycobacterium tuberculosis* (MT) infects the pulmonary parenchyma weeks or months prior to the onset of NMOSD signs [13]. The pathophysiological relationship between PT and NMOSD is not yet established.

Herein, a fatal case of concomitant diagnosis of seropositive NMOSD and PT in a young Tunisian male patient was reported. The hypothesized immunological disturbances and the adequate therapeutic attitudes were also discussed.

## Case report

A 28-year-old Tunisian male patient, from a rural area, with alcohol consumption and occasional cannabis smoking habits and with no medical history, presented to our department. The family history was not contributory. He was admitted with complaints of sudden onset of lower limbs weakness and dysuria appearing 7 days prior to admission. Dry cough, night sweat, and anorexia with weight loss preceded the neurological signs by 2 months. These symptoms did not improve after ambulatory care with broad-spectrum antibiotics. No visual complaints were reported by the patient.

The patient was febrile and fully conscious. Pulmonary auscultation revealed bilateral crepitations. He was tachycardic. Neurological examination revealed complete paraplegia and decreased deep tendon reflexes. The Babinski sign was present. He had a T4 sensory level and urinary retention.

Blood tests showed the presence of neutrophilic leukocytosis (total white cell 15 × 10^9^/l, neutrophils 11.8 × 10^9^/l) and elevated C-reactive protein serum levels (62 mg/l). The serological markers of infection with hepatitis viruses B and C, and human immunodeficiency virus (HIV), were negative.

**Fig. 1:**
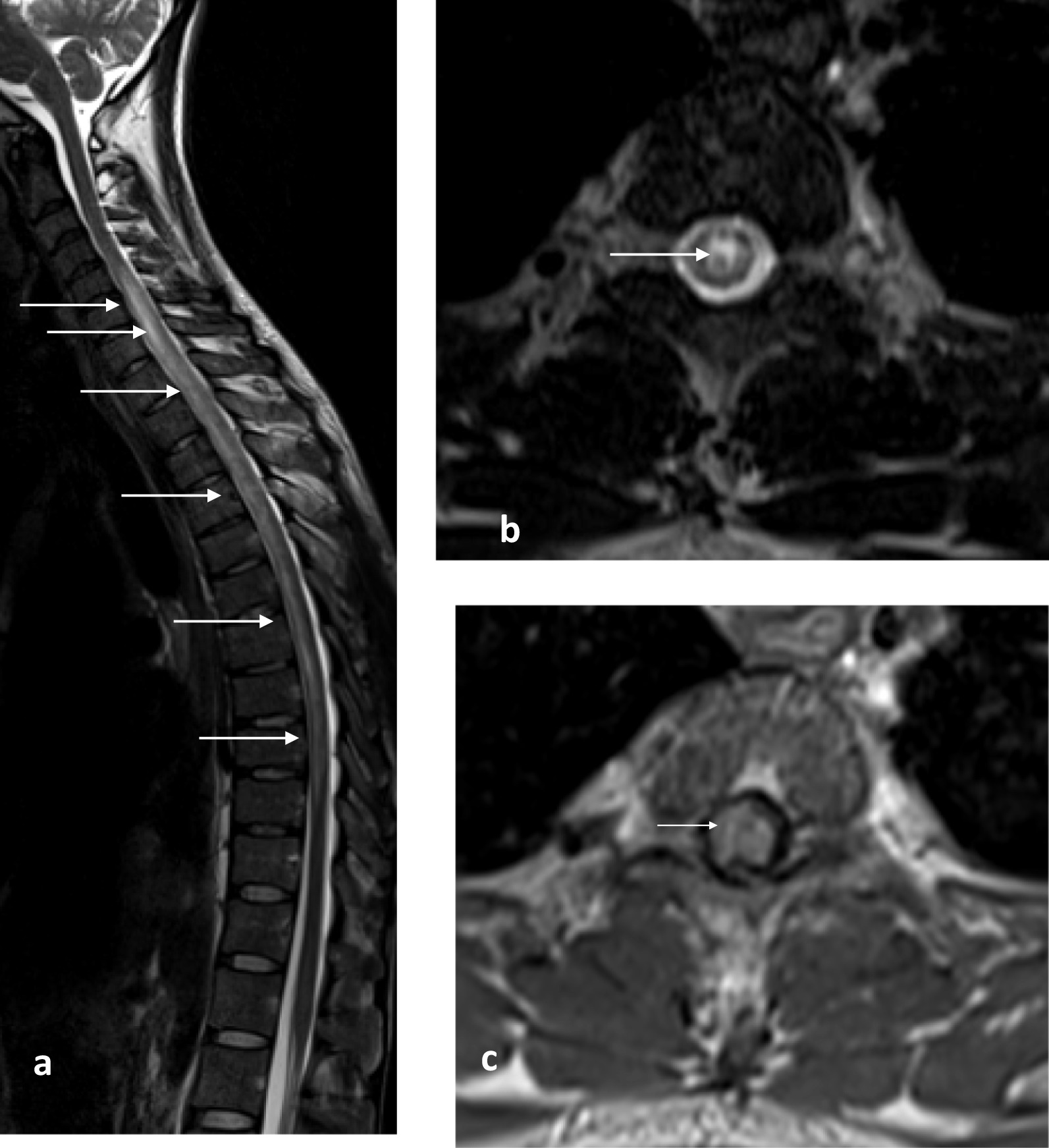
**a**–**c** Magnetic resonance images of the spinal cord. **a** Sagittal T2-weighted image showing longitudinally extensive hyperintensity, extending from C5 to T10 level (arrows); **b** axial T2-weighted image showing hyperintensity in the cervical cord involving gray matter and appearing as a bright spotty lesion (arrow); **c** axial T1 post-contrast administration weighted image showing heterogeneous spinal cord enhancement (arrow)

**Fig. 2:**
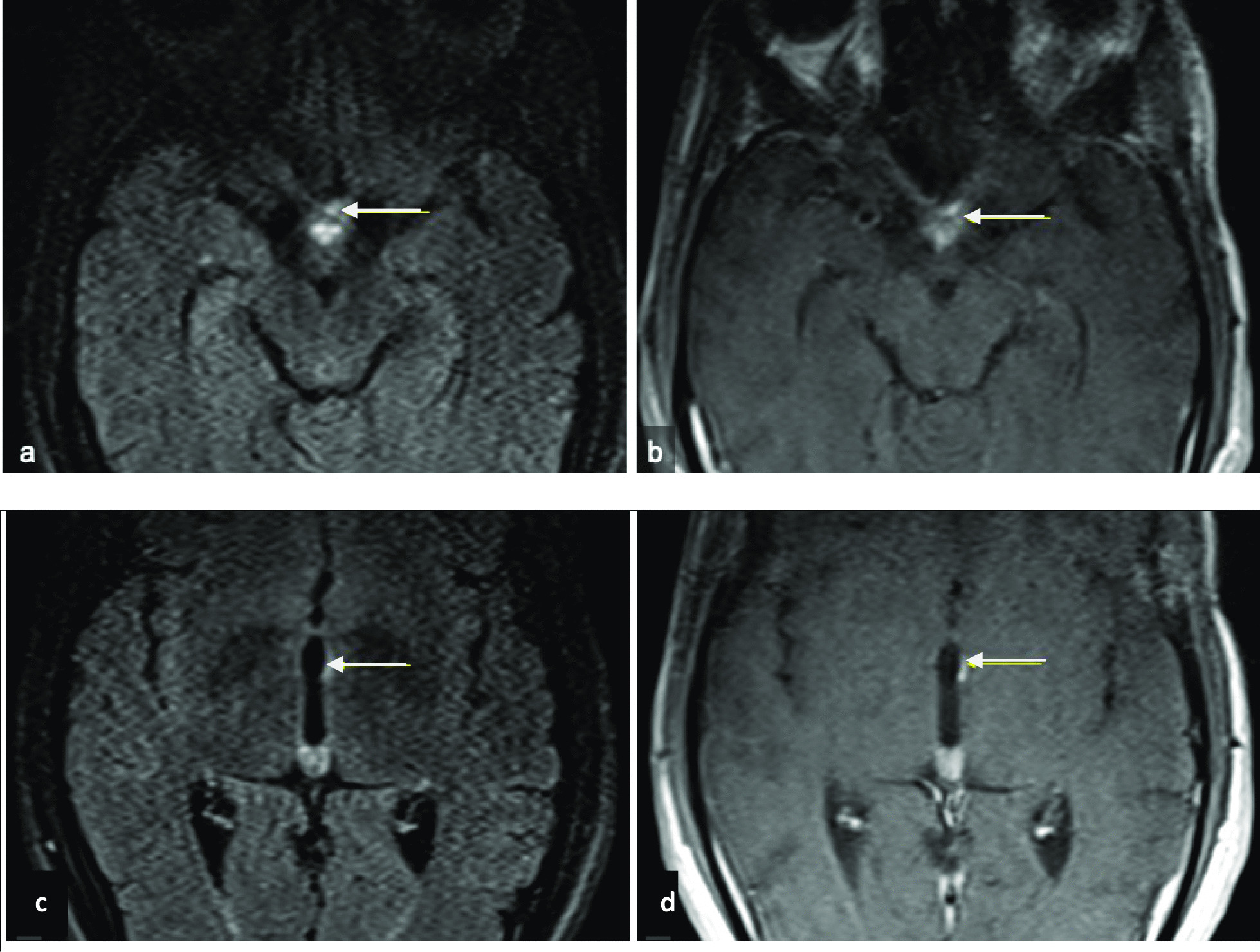
**a**–**d** Brain magnetic resonance images. **a** Axial FLAIR-weighted image showing hyperintensity of the optic chiasma (arrow); **b** axial T1 post-contrast administration weighted MRI image showing enhancement of the optic chiasma extending to the right optic nerve (arrow); **c** axial FLAIR-weighted image showing a focal hyperintensity of the left wall of the third ventricle (arrow); **d** axial T1 post-contrast administration weighted image showing enhancement of the left wall of the third ventricle (arrow)

Magnetic resonance imaging (MRI) of the spine showed an intramedullary tumefactive T2 hyperintensity extending from C5 to T10, which was consistent with the diagnosis of LETM (Fig. 1a). Hyperintensity mostly involved the gray matter, as seen in the transverse plane. The presence of T2 bright spotty lesions was demonstrated (Fig. 1b). Administration of a contrast agent revealed a multifocal and heterogeneous enhancement (Fig. 1c). Brain MRI showed T2 hyperintensity of the optic chiasma (Fig. 2a) and an abnormal enhancement of the right optic nerve as well as the optic chiasma (Fig. 2b). It also revealed hyperintensity and enhancement of the left wall of the third ventricle, in the hypothalamus (Fig. 2c, d). No tuberculomas, leptomeningeal enhancement, or vertebral lesions were observed.

Cerebrospinal fluid (CSF) analysis revealed mild pleocytosis (7 lymphocytes/mm^3^), hyperproteinorachia (3.23 g/l), and hypoglycorrhachia (1.9 mmol/l). Qualitative assessment of the intrathecal IgG synthesis in the CSF by isoelectric focusing did not show abnormalities. Serum anti-AQP4ab was detected with a cell-based assay using transfected cells. Diagnosis of NMOSD was retained according to the consensus criteria [3].

**Fig. 3:**
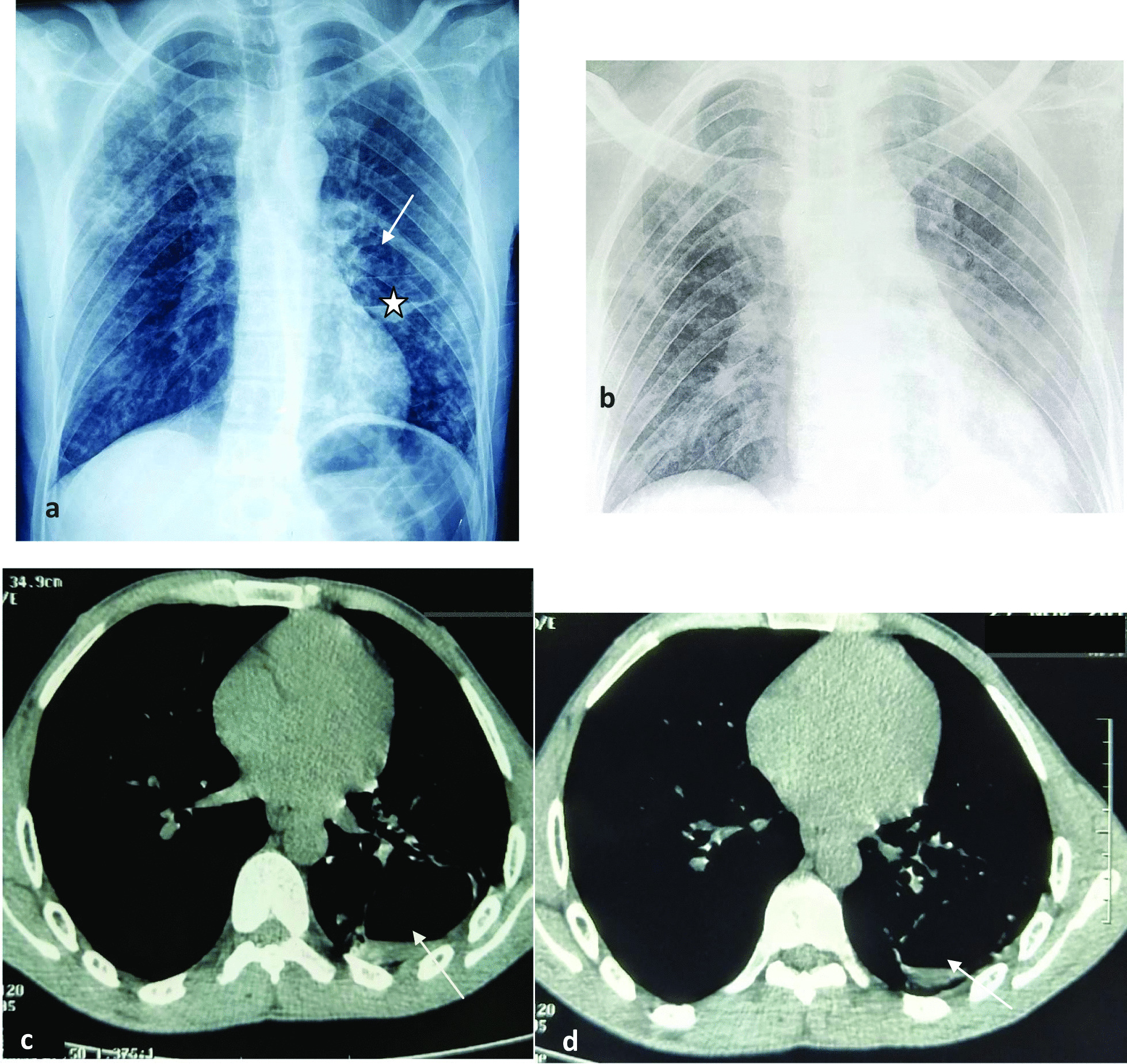
**a**–**d** Anteroposterior chest X-ray and chest CT scan demonstrating diffuse reticulonodular infiltrates in both lungs and left pulmonary excavations. **a** Initial anteroposterior chest X-ray on admission illustrating pulmonary excavation (arrow) with air-fluid level (star). **b** Follow-up chest X-ray, performed 3 months after antituberculosis therapy initiation, showing regression of excavation. **c**, **d** CT scan (mediastinal window), performed on admission, showing pulmonary excavation with air-fluid level (arrow)

Additional investigation using chest-X-ray and CT scan test demonstrated typical radiological signs for PT, consisting of pulmonary excavation with air-fluid level (Fig. 3a, c, d). The tuberculin skin test was positive. To search for MT in the pulmonary system, both acid-fast bacilli test and sputum culture were negative.

As our region is endemic for MT and the radiological findings were typical with a positive tuberculin skin test, diagnosis of smear-negative PT coincident with NMOSD was retained. Investigation for MT by polymerase chain reaction (PCR) in the CSF was negative.

Antituberculosis treatment (AT) (isoniazid, rifampicin, pyrazinamide, and ethambutol) was initiated*.* Methylprednisolone intravenous pulses were administered together with AT*.*

On the fourteenth day of treatment, intravenous immunoglobulins (IVIG) were administered. The patient became apyretic, and his food intake improved, but the lower limbs weakness persisted.

Three months after the first onset of the neurological symptoms, the patient was readmitted for fever, chills, hypotension, and burning urination. At that time, pulmonary symptoms improved. There was some radiological improvement as the follow-up chest radiography demonstrated the disappearance of the air-fluid level (Fig. 3b). However, a residual paraplegia was noted. Diagnosis of severe sepsis having urinary source was retained. A specific antibiotherapy was introduced in addition to AT with a control of the infectious process. However, 7 days later, the patient presented with a complaint of rapid right blurring of vision. His visual acuity in the right eye was limited to finger counting. A relative afferent pupillary defect and optic disc pallor in the right eye were noted. Fundoscopy of the left eye showed a normal appearance of the optic disc. Diagnosis of right retrobulbar ON was therefore made. The patient was treated with repeated courses of corticosteroids but with no improvement in the visual acuity. One month later, the patient died, suddenly, at home.

## Discussion

We report a case with concomitant diagnosis of NMOSD and smear-negative PT. NMOSD first manifested by LETM occurring 2 months after first pulmonary symptoms. Microbiologically, it was not possible to confirm the presence of MT. In fact, it is already known that MT is difficult to confirm microbiologically with sputum smear or culture specimens given its paucibacillary nature [14]. Moreover, half of all the cases of PT are smear negative [15]. Lack of response to nonspecific antibiotics and initial positive response to AT supported our diagnosis. Furthermore, biological findings in CSF argued against CNS TP involvement in our patient. First, mild pleiocytosis is not in line with the cellular formula found in the majority of cases with CNS involvement with a higher formula ranging between 100 and 500/mm [16]. Secondly, glycorrhachia was only slightly decreased (1.9 mmol/l), and finally, MT-PCR on CSF was negative. On the other hand, CNS-MRI study did not show any sign in favor of infected intervertebral disc or bone destruction towards the diagnosis of Pott’s disease. Also, no leptomeningeal enhancement on the spinal cord or on the brain and no tuberculoma were observed. All these arguments together support the diagnosis of concomitant diseases (NMOSD and PT) in our patient.

Retrobulbar ON diagnosed shortly after sepsis was most likely a second relapse of NMOSD. Indeed, papillitis, neuroretinitis, or optic nerve tuberculoma, known to be the most common clinical presentation of TB optic neuropathy [17], were not found in our patient. Our patient had no posterior or panuveitis, which is an additional ocular comorbidity reported in nearly 90% of tuberculous ON [17]*.* Finally, in CNS PT, ON mostly results from chronic papilledema due to meningitis, which was not the case in our patient [8]. Toxic neuritis due to isoniazid or ethambutol was also ruled out since ON was unilateral in our case.

What makes our work different from earlier reported cases is the simultaneous identification of MT and NMOSD. The clinical signs appeared with a short time interval, but diagnosis of the two conditions was made at the same time. In a study by Brey and Henning [18], a young male patient was reported to exhibit two NMOSD relapses with negative AQP4ab, preceded a few weeks earlier by a documented PT relapse. A Chinese study [5] reported two cases presenting LETM as a first manifestation of NMOSD. LETM coincided with a past history of PT.

On the other hand, the association of NMOSD and PT with central nervous system dissemination was also previously described [4, 12]. Severity of relapses is a common clinical feature for NMOSD, in both coincident or associated MT infection.

Current clinical data are conflicting and insufficient to establish a confirmed association between PT and NMOSD. A South African study [13] found an increased odds ratio (OR = 4.6) for the presence of PT in NMOSD patients versus a control group, and in a Chinese controlled study, AT was shown to reduce disability and relapses in 13 patients with steroid-refractory NMOSD [19]. Another study conducted in 2014 by Li *et al.* in the same region [20] failed to demonstrate any association between the two entities.

According to Ren Z. *et al.* [21], a mechanism of an infection-induced cross-reactivity due to MT is suggested. MT may induce lymphocytes sensitization against self-proteins, such as AQP4. Subsequent entry of self-reactive lymphocytes into the CNS could impair immunological tolerance and then produce astrocyte damage [22].

The therapeutic approach of concomitant NMOSD and PT is challenging. In fact, the immunosuppressive agents used in patients with NMOSD can lead to tuberculosis dissemination [22]. This is particularly important for long-term management, since prolonged use of high doses of steroids is associated with an increased risk of MT infection or reactivation [23]. Nevertheless, in our patient, clinical worsening was unlikely related to PT dissemination after immunosuppressive therapy. At the 3-month evaluation, no clinical signs suggestive of tuberculous meningitis, encephalopathy (stiff neck, behavior changes, or seizures) or Pott’s disease (radiating rood pain, palpable mass) were observed [24, 25]. The sudden death at home is thought to be related to decubitus complications, such as pulmonary embolism.

In the earlier reported cases, including a case of one patient with seronegative form of NMOSD, combination of corticotherapy and AT was the most adopted therapeutic strategy [4, 5, 10, 12]. Some authors have suggested that plasma exchange or IVIG may be an alternative option to ovoid MT dissemination [22]. Indeed, the use of IVIG was a safe option in our case.

For long-term immunosuppressive therapy, there is also a lack of available data. The therapeutic choice is difficult because azathioprine is contraindicated in latent or active PT, and cyclophosphamide cannot be used in association with AT [23].

Recently, a case of NMOSD manifesting during the initial phase of AT in a female patient was treated with rituximab in cyclic infusions with a favorable outcome [9].

On the other hand, mitoxantrone should be given more attention in such concomitant conditions. In multiple sclerosis, a common differential diagnosis of NMOSD, mitoxantrone was proposed for patients with latent infection or those at risk of MT infection [23] since its mechanism of action inhibits MT exponential growth.

## Conclusions

In summary, MT infection should be considered by the practitioner as a possible additional condition to the neurological signs of NMOSD. MT infection can be concomitant or associated with NMOSD. This is important, especially in high-burden settings. Our work as well as earlier reports reinforce the hypothesis that MT infection is a possible infectious environmental factor involved in the development of NMOSD, even in the absence of CNS invasion by MT. A better understanding of the mechanism by which MT exerts its pathological effect, contributing to astrocyte damage, can be helpful for the clarification of the therapeutic approach of this complex association.

## Data Availability

Not applicable.

## Abbreviations

NMOSD: Neuromyelitis optica spectrum disease
ON: Optic neurotis
LETM: Longitudinally extensive transverse myelitis
Anti-AQP4ab: Anti-aquaporin-4 autoantibodies
MT: *Mycobacterium tuberculosis*
PT: Pulmonary tuberculosis
CNS: Central nervous system
MRI: Magnetic resonance imaging
CSF: Cerebrospinal fluid
PCR: Polymerase chain reaction
AT: Antituberculosis treatment
IVIG: Intravenous immunoglobulins
